# Interaction between membranous EBP50 and myosin 9 as a favorable prognostic factor in ovarian clear cell carcinoma

**DOI:** 10.1002/1878-0261.13503

**Published:** 2023-08-30

**Authors:** Mayu Nakagawa, Toshihide Matsumoto, Ako Yokoi, Miki Hashimura, Yasuko Oguri, Ryo Konno, Yu Ishibashi, Takashi Ito, Kensuke Ohhigata, Yohei Harada, Naomi Fukagawa, Yoshio Kodera, Makoto Saegusa

**Affiliations:** ^1^ Department of Pathology Kitasato University School of Medicine Sagamihara Japan; ^2^ Department of Pathology Kitasato University School of Allied Health Science Sagamihara Japan; ^3^ Center for Disease Proteomics, School of Science Kitasato University Sagamihara Japan

**Keywords:** cancer stem cell, EBP50, epithelial–mesenchymal transition, migration, myosin 9, ovarian clear cell carcinoma

## Abstract

Ezrin‐radixin‐moesin‐binding phosphoprotein 50 (EBP50) is a scaffold protein that is required for epithelial polarity. Knockout (KO) of membranous EBP50 (Me‐EBP50) in ovarian clear cell carcinoma (OCCC) cells induced an epithelial–mesenchymal transition (EMT)‐like phenotype, along with decreased proliferation, accelerated migration capability, and induction of cancer stem cell (CSC)‐like properties. Shotgun proteomics analysis of proteins that co‐immunoprecipitated with EBP50 revealed that Me‐EBP50 strongly interacts with myosin 9 (MYH9). Specific inhibition of MYH9 with blebbistatin phenocopied Me‐EBP50 KO, and blebbistatin treatment potentiated the effects of Me‐EBP50 KO. In OCCC cells from clinical samples, Me‐EBP50 and MYH9 were co‐localized at the apical plasma membrane. Patients with a combination of Me‐EBP50‐high and MYH9‐high scores had the best prognosis for overall and progression‐free survival. Our data suggest that Me‐EBP50 has tumor‐suppressive effects through the establishment and maintenance of epithelial polarization. By contrast, loss of Me‐EBP50 expression induces EMT‐like phenotypes, probably due to MYH9 dysfunction; this results in increased cell mobility and enhanced CSC‐like properties, which in turn promote OCCC progression.

## Graphical abstract

In ovarian clear cell carcinoma (OCCC), membranous Ezrin‐radixin‐moesin‐binding phosphoprotein 50 (EBP50) has tumor‐suppressive effects through the establishment and maintenance of epithelial polarization. By contrast, loss of membranous EBP50 expression induces epithelial–mesenchymal transition‐like phenotypes, probably due to myosin 9 dysfunction; this results in increased cell mobility and enhanced cancer stem cell‐like properties, which in turn promote OCCC progression.

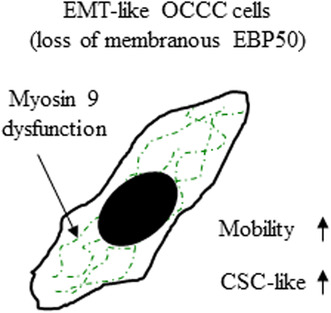

**Abbreviations**
CSC: cancer stem cell
Cyt‐EBP50: cytoplasmic EBP50
EBP50: Ezrin‐radixin‐moesin‐binding phosphoprotein 50
EMT: epithelial–mesenchymal transition
IHC: immunohistochemistry
Me‐EBP50: membranous EBP50
MYH9: myosin 9
OCCC: ovarian clear cell carcinoma
PDZ: cytosolic PSD‐95/*Drosophila* discs large/ZO‐1

## 1 Introduction

Epithelial ovarian carcinoma (EOC) is the seventh most commonly diagnosed tumor among women worldwide and carries the highest mortality rate of all gynecological malignancies [1, 2, 3]. Recently, EOC has been categorized into two types based on clinical, cellular, and molecular characteristics [4]. Type I tumors (which are clinically indolent and genetically stable) include low‐grade serous, low‐grade endometrioid, clear cell, and mucinous carcinomas [5, 6]. Type II tumors are more aggressive and genetically unstable; malignancies in this group include high‐grade serous, high‐grade endometrioid, and some clear cell carcinoma and carcinosarcomas [7, 8].

Ovarian clear cell carcinoma (OCCC) is recognized as a distinct entity among EOC for the following reasons. First, OCCC accounts for 5–10% of all EOC in North America and 12% in western countries; its prevalence is higher in East Asia, where it accounts for 25–30% and 10–12% of all EOC in Japan and Korea, respectively [9, 10, 11]. Second, endometriosis, as well as adenofibroma, are considered precursor lesions of OCCC, as women with endometriosis have a 3‐fold increased risk of developing OCCC compared with those without such lesions [12]. Third, the majority of patients with OCCC are diagnosed at a younger age and an earlier stage (66.4% of patients had stage I disease [13]), the tumors rarely occur bilaterally, and they are often associated with complications of thromboembolism and hypercalcemia [14]. By contrast, some patients with advanced OCCC have a significantly worse prognosis than patients with advanced high‐grade serous carcinoma, probably due to a propensity to develop chemoresistance; this contrasts with non‐OCCCs, which are relatively sensitive to chemotherapy [15].

Ezrin‐radixin‐moesin‐binding phosphoprotein 50 (EBP50), which is also known as Na^+^/H^+^ exchanger regulatory factor 1, is a 55‐kDa phosphoprotein and a member of the family of cytosolic PSD‐95/Drosophila discs large/ZO‐1 (PDZ) adaptor proteins [16]. EBP50 is highly expressed in several epithelial tissues and localized at apical plasma membrane of polar epithelia, where it regulates apical microvilli formation [17, 18]. Alterations in the apical localization of EBP50 contribute to tumor progression due to the disruption of epithelial morphology in several human malignancies [19, 20, 21]. In addition, estrogen receptor‐α‐dependent EBP50 expression negatively regulates trans‐differentiation toward the morular phenotype in endometrial carcinoma cells through interacting with PTEN (phosphatase and tensin homolog deleted on chromosome 10) and β‐catenin [22].

Here, we hypothesized that the role of membranous EBP50 (Me‐EBP50) in establishment and maintenance of epithelial polarity might have prognostic significance in OCCC. This hypothesis was partly based on observation that cytoplasmic EBP50 (Cyt‐EBP50) is closely associated with a poor prognosis in the tumors [23]. To test this, we investigated the functional role of Me‐EBP50 in modulating cell proliferation and mobility in OCCC cells. We identified myosin 9 (MYH9) as a Me‐EBP50 binding partner, and found that the Me‐EBP50/MYH9 complex plays an important role in determining the biological behavior of OCCC.

## 2 Materials and methods

### 2.1 Plasmids and cell lines

Glutathione S‐transferase (GST)‐fusion protein constructs including full length, PDZ1, PDZ2, and EB domains were used as described previously [23].

Two OCCC cell lines, OVISE (RRID:CVCL_3116) and TOV‐21G (RRID:CVCL_3613), were obtained from the National Institute of Biomedical Innovation (Osaka, Japan) and the American Type Culture Collection (Manassas, VA, USA), respectively. They were used within 6 months of thawing and were periodically authenticated by monitoring of cell morphology and growth curve analysis. All experiments were performed with mycoplasma‐free cells. The Me‐EBP50‐knockout (KO) cell line was generated using OVISE cells (which have high Me‐EBP50 expression). Briefly, the guide RNA sequence (gRNA: 5′‐GAGAAGGGTCCGAACGGCTACGG‐3′) was designed using CRISPRdirect (https://crispr.dbcls.jp/: last accessed on 6 June 2021). The complementary oligonucleotides for gRNA were annealed and cloned into pSpCas9n(BB)‐2A‐Puro (PX459) V2.0 (Addgene #62988, Watertown, MA, USA). The pSpCas9n(BB)‐2A‐Puro (PX459) V2.0/gRNA construct was transfected into OVISE cells to establish Me‐EBP50‐KO cell lines. Spindle‐shaped cells were defined as those that showed narrow and elongated phenotypes, along with weak or absent intercellular adhesion.

### 2.2 Clinical cases

A total of 120 OCCC cases, surgically resected at Kitasato University Hospital between 2005 and 2019, were selected from our patient records according to the criteria of the 2014 World Health Organization classification and the TNM classification [24, 25]. All patients underwent oophorectomy with or without hysterectomy. No patients had received paclitaxel/carboplatin‐based chemotherapy before surgical treatment, whereas most patients had been treated with chemotherapy after surgical resection. All cases showed complete resection of the tumors, and no cases had residual tumors after debulking surgery. All tissues were routinely fixed in 10% formalin and processed for embedding in paraffin wax. Approval for this study was given by the Ethics Committee of the Kitasato University School of Medicine (B20‐181). The study methodologies conformed to the standards set by the Declaration of Helsinki, and the experiments were undertaken with the understanding and written consent of each subject.

### 2.3 Shotgun proteomics analysis

Shotgun proteomics using proteins that co‐immunoprecipitated with EBP50 was performed as described previously [23, 26]. For co‐immunoprecipitation, anti‐EBP50 antibody was bound and crosslinked to Protein G Dynabeads (Invitrogen, Carlsbad, CA, USA) according to the manufacturer's instructions. Cells were lysed with IP buffer [10 mm Tris–HCl (pH 7.5), 100 mm NaCl, 1% Nonidet P‐40], and the resulting supernatant was incubated with EBP50 antibody‐conjugated Protein G Dynabeads in lysate solution. The beads were washed before recovery of co‐immunoprecipitated materials. Quantitative analysis of shotgun proteomics data was achieved by spectral counting as described previously [23].

### 2.4 Antibodies and reagents

Anti‐Ezrin/radixin/moesin (ERM), anti‐phospho (p) Ezrin (at Thr567)/radixin (at Thr564)/moesin at Thr558) (pERM), anti‐poly (ADP‐ribose) polymerase 1 (PARP1), and anti‐vimentin antibodies were purchased from Cell Signaling Technology (Danvers, MA, USA). Anti‐EBP50, anti‐β‐actin, anti‐MYH9 (mouse), anti‐vinculin, and anti‐Sox2 antibodies were obtained from Abcam (Cambridge, MA, USA). Anti‐E‐cadherin, anti‐p21^waf1^, and anti‐cyclin D1 antibodies were from Dako (Copenhagen, Denmark). Anti‐cyclin A2 and anti‐cyclin E antibodies were from Novocastra (Newcastle, UK). Anti‐p27^kip1^, anti‐aldehyde dehydrogenase 1 (ALDH1), and anti‐N‐cadherin antibodies were from BD Biosciences (San Jose, CA, USA). Anti‐MYH9 (Rabbit) and anti‐cyclin B1 antibodies were purchased from Proteintech (Rosemont, IL, USA) and Santa Cruz Biotech (Santa Cruz, CA, USA), respectively. Blebbistatin and MG132 were obtained from Toronto Research Chemicals (North York, ON, Canada) and Sigma‐Aldrich Chemicals (St. Louis, MO, USA), respectively.

### 2.5 GST pull‐down assay

GST‐EBP50‐full length, GST‐EBP50‐PDZ1, GST‐EBP50‐PDZ2, and GST‐EBP50‐EB were induced by 1 mm isopropyl‐β‐d‐thiogalactopyranoside and purified with glutathione‐Sepharose beads. OVISE cell lysates were mixed with purified GST‐EBP50‐full length, GST‐EBP50‐PDZ1, GST‐EBP50‐PDZ2, or GST‐EBP50‐EB immobilized on the beads. Pull‐down assays were performed at 4 °C overnight. The beads were then washed thoroughly with wash buffer [10 mm Tris–HCl (pH 7.5), 150 mm NaCl, 1 mm EDTA, and 1% Nonidet P‐40]. Bound proteins were eluted by boiling in SDS/PAGE loading buffer, separated by SDS/PAGE, and detected by immunoblotting and Coomassie Brilliant Blue staining as described previously [23].

### 2.6 Western blot assays

Total cellular proteins were isolated using RIPA buffer (20 mm Tris–HCl [pH 7.2], 1% Nonidet P‐40, 0.5% sodium deoxycholate, 0.1% sodium dodecyl sulfate). The cytoplasmic, membranous, and nuclear fractions were prepared using ProteoExtract Subcellular Proteome Extraction kit (Merck KGaA, Darmstadt, Germany). Aliquots of the proteins were resolved by SDS/PAGE, transferred to PVDF membranes, and probed with primary antibodies coupled to the ECL detection system (GE Healthcare, Buckinghamshire, UK) as described previously [22, 23, 26, 27].

### 2.7 Flow cytometry and Aldefluor assay

Cells were fixed using 70% alcohol and stained with propidium iodide (Sigma) for cell cycle analysis. ALDH1 enzyme activity in viable cells was determined using a fluorogenic dye‐based Aldefluor assay (Stem Cell Technologies, Grenoble, France) according to the manufacturer's instructions. The prepared cells were analyzed by flow cytometry using BD FACS Calibur (BD Biosciences) and cellquest pro software version 3.3 (BD Biosciences) as described previously [22, 23, 26, 27].

### 2.8 Spheroid assay

Cells (×10^3^) were plated in low cell binding plates (Thermo Fisher Scientific, Yokohama, Japan) in Cancer Stem Cell Premium (ProMab Biotech, Richmond, CA, USA). Uniform spheroids of at least 50 μm in diameter were counted approximately 2 weeks after plating as described previously [22, 27].

### 2.9 Immunohistochemistry (IHC)

IHC was performed using a combination of the microwave oven heating and polymer immunocomplex (Envision, Dako) methods as described previously [23, 26].

For evaluation of IHC findings, scoring of membranous, cytoplasmic, or nuclear immunoreactivity for EBP50, E‐cadherin, Snail, ALDH1, vimentin, and Sox2 was performed on the basis of the percentage of immunopositive cells and the immunointensity with multiplication of the values of the two parameters as described previously [23, 26]. For EBP50, cases were defined as Me‐ or Cyt‐immunopositive when the predominant immunoreactions were observed in the membrane or cytoplasmic components. To examine the prognostic significance of Me‐EBP50, Cyt‐EBP50, and MYH9, the scores were divided into two categories (high and low) on the basis of the mean values as the cutoff.

### 2.10 Immunofluorescence

The slides of clinical samples were heated in 10 mm citrate buffer (pH 6.0) for 3 × 5‐min cycles using a microwave oven and then incubated overnight with anti‐EBP50 (rabbit) and anti‐MYH9 (mouse) antibodies. Alexa 488 and 570 (Thermo Fisher Scientific, Waltham, MA, USA) were used as secondary antibodies and analyzed with a BZ‐X700 microscope (KEYENCE Co., Osaka, Japan). To detect focal adhesions (FAs), OV‐EBP‐KO and mock cells on slides were stained with anti‐vinculin primary and fluorescein goat anti‐rabbit secondary antibodies. Vinculin was chosen, because there were no significant differences in the measurement of morphological parameters from staining with various anti‐FA antibodies including vinculin, focal adhesion kinase, paxillin, and zyxin [27]. The numbers of immunopositive sites at the peripheries of cells were calculated per unit area determined by imagej software version 1.41 (NIH, Bethesda, MD, USA; http://imageJ.nih.gov/ij) as described previously [22, 23].

### 2.11 Proximity ligation assay (PLA)

The slides were heated in 10 mm Tris‐EDTA buffer (pH 9.0) for 3 × 5 min cycles using a microwave oven and then incubated overnight with primary antibodies. The following antibody pairs were used in combination of EBP50 (rabbit)/MYH9 (mouse) or alone as negative control. After washing, the slides were treated according to the manufacturer's protocol using the Duolink Detection kit with PLA PLUS and MINUS probes for mouse and rabbit (Olink Bioscience, Uppsala, Sweden) as described previously [26].

### 2.12 Wound‐healing assay

Cells were seeded into 24‐well tissue culture plates and grown to reach almost total confluence. After a cell monolayer formed, a wound was scratched with a sterile 200‐μL tip. The area of the wound was analyzed using imagej software version 1.41. Cell migration parameters were calculated in pixels as wound closure as described previously [22, 27].

### 2.13 Migration assay

Cell migration was determined using 24‐well Transwell chambers with 8‐μm pore size (Corning, NY, USA). The lower chamber was filled with medium containing 10% serum. Cells were suspended in serum‐free upper medium with or without blebbistatin and seeded into the upper chamber. After 24 h, the number of cells stained by hematoxylin–eosin (HE) on the bottom surface of the polycarbonate membranes was counted visually using a light microscope as described previously [22, 27].

### 2.14 Statistics

Comparative data were analyzed using the Mann–Whitney *U*‐test and Spearman's correlation coefficient. Overall survival (OS) was calculated as the time between onset and death or the date of the last follow‐up evaluation. Progression‐free survival (PFS) was also examined from the onset of treatment until relapse, disease progression, or last follow‐up evaluation. Overall survival and PFS were estimated using the Kaplan–Meier methods, and the statistical comparisons were made using the log‐rank test. Univariate and multivariate analyses were also performed using the Cox proportional hazards regression model. The cutoff for statistical significance was set as *P* < 0.05.

## 3 Results

### 3.1 Prognostic significance of Me‐ and Cyt‐EBP50 in OCCC

**Fig. 1:**
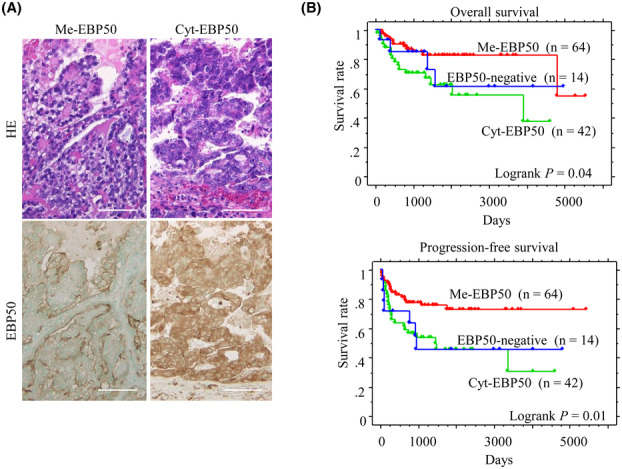
Relationship between EBP50 expression and prognosis in ovarian clear cell carcinoma (OCCC). (A) Staining with HE (upper) and immunohistochemistry (IHC) for EBP50 (lower) in OCCC. Note the apical plasma membranous (Me)‐ and cytoplasmic (Cyt)‐EBP50 immunoreactivities (left and right). A total of 120 OCCC cases were immunohistochemically investigated. Original magnification, ×200. Scale bar = 50 μm. (B) OS (upper) and PFS (lower) relative to EBP50 (Me‐ versus Cyt‐ versus negative‐EBP50 expression). *n*, number of cases.

Representative IHC findings for Me‐ and Cyt‐EBP50 in OCCC are illustrated in Fig. 1A. EBP50 immunoreactivities were observed in apical plasma membrane or cytoplasmic components of OCCC cells. Predominant Me‐ and Cyt‐EBP50 and negative immunoreactivities were observed in 64 (53.3%), 42 (35%), and 14 (11.7%) of 120 OCCC cases, respectively. Kaplan–Meier analysis showed that patients with Me‐EBP50 immunoreactivity had a favorable prognosis with regard to OS and PFS when compared with patients with Cyt‐EBP50 and the negative immunoreactions (Fig. 1B). These findings suggest that there is a close association between subcellular distribution of EBP50 and tumor behavior in OCCC.

### 3.2 Knockout of Me‐EBP50 is associated with decreased proliferation and accelerated mobility of OCCC cells

In OCCC cell lines, there were no differences in expression of MYH9, E‐cadherin, and Snail between OVISE and TOV‐21G cells, when the cells were rendered quiescent by serum starvation and were subsequently stimulated with serum. By contrast, increased expression of Sox2 and N‐cadherin and decreased expression of EBP50, ALDH, and vimentin were evident in OVISE as compared to TOV‐21G cells (Fig. S1A). In addition, migration capacity was significantly higher in the former as compared to the latter (Fig. S1B).

**Fig. 2:**
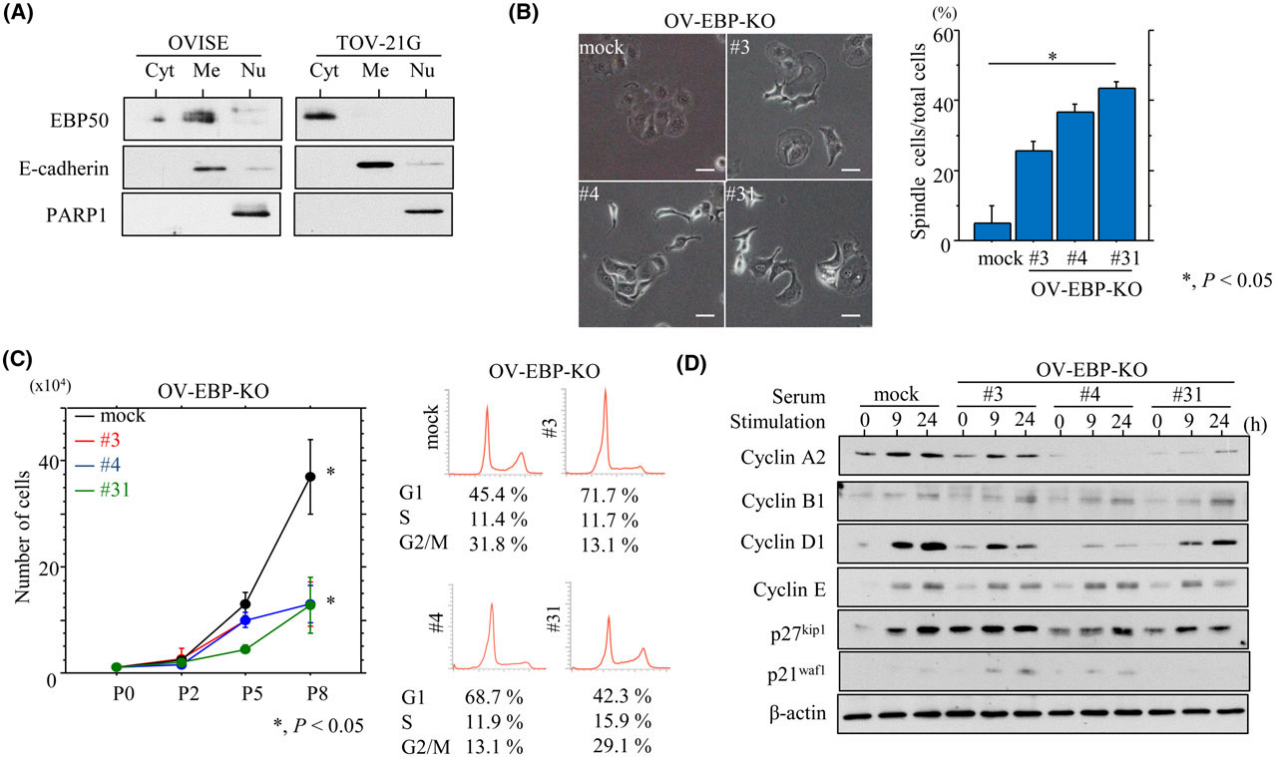
Changes in cell morphology and proliferation following membranous EBP50 (Me‐EBP50) knockout (KO) in ovarian clear cell carcinoma (OCCC) cells. (A) Western blot analysis for the indicated proteins in cytoplasmic (Cyt), membranous (Me), and nuclear (Nu) fractions from OVISE (left) and TOV‐21G (right). Note the predominant Me‐EBP50 in OVISE cells, in contrast to the predominant cytoplasmic EBP50 (Cyt‐EBP50) in TOV‐21G cells. The experiments were performed in triplicate. (B) Left: phase contrast images of OV‐EBP‐KO cells. Note the alterations in cell morphology toward fibroblastic appearances. Scale bar = 10 μm. Right: the percentages of spindle‐shaped relative to total cells are presented as means ± SDs. The experiments were performed in triplicate. Statistical analyses were carried out using the Mann–Whitney *U*‐test. *, *P* < 0.05. (C) Left: three independent OV‐EBP‐KO and mock cell lines were seeded at low density. The cell numbers are presented as means ± SDs. P0, P2, P5, and P8 are 0, 2, 5, and 8 days after seeding, respectively. Right: flow cytometry analysis of OV‐EBP‐KO and mock cells 3 days after seeding. The experiments were performed in triplicate. Statistical analyses were carried out using the Mann–Whitney *U*‐test. *, *P* < 0.05. (D) Western blot analysis for the indicated proteins in total lysates from OV‐EBP‐KO and mock cells following re‐stimulation of serum‐starved (24 h) cells with 10% serum for the indicated times. The experiments were performed in triplicate.

EBP50 expression was predominantly membranous or cytoplasmic in OVISE and TOV‐21G cells, respectively (Fig. 2A), as described previously [23]. To examine the functional role of Me‐EBP50, we first established three independent Me‐EBP50 KO OVISE cell line clones (OV‐EBP‐KO#3, #4, and #31). The OV‐EBP‐KO cells demonstrated a significant switch toward a fibroblastic morphology, known as epithelial–mesenchymal transition (EMT)‐like features, as compared to mock cells (Fig. 2B).

To examine whether Me‐EBP50 KO affects proliferation, the three independent OV‐EBP‐KO cell lines were seeded at low density. The OV‐EBP‐KO cells tended to proliferate more slowly as compared to mock cells, particularly in the exponential growth phase; there were also proportionally more cells in G1 phase and less cells in G2/M phase (Fig. 2C). To further examine alterations in the expression of several cell cycle‐related molecules during cell growth, the OV‐EBP‐KO cells were rendered quiescent by serum starvation and were subsequently stimulated with serum. At 9 and 24 h after release into the cell cycle, the levels of cyclin A2 and cyclin D1 were lower in OV‐EBP‐KO cells relative to the mock cells, whereas the expression of cyclin B1, cyclin E, p21^waf1^, and p27^kip1^ was unchanged (Fig. 2D).

**Fig. 3:**
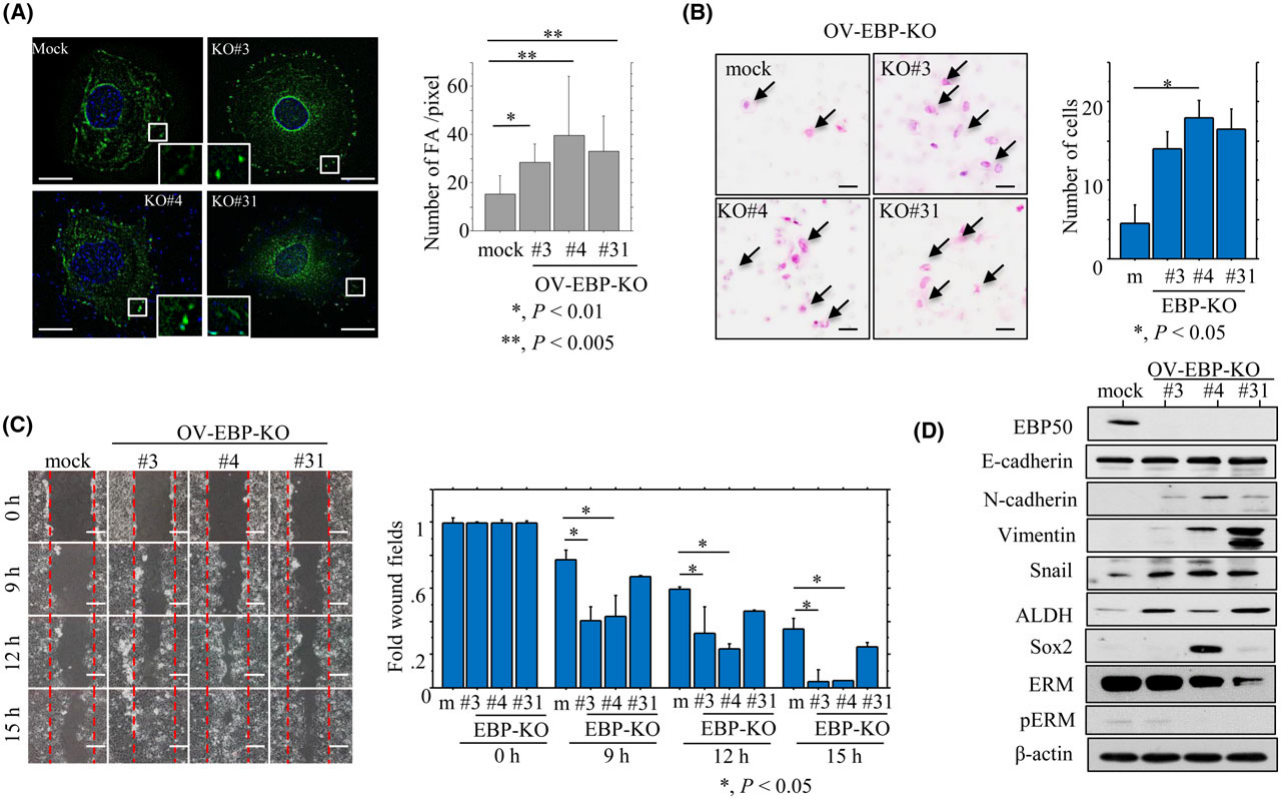
Relationship between membranous EBP50 (Me‐EBP50) knockout (KO) and cell mobility. (A) Left: OV‐EBP‐KO and mock cells were stained with anti‐vinculin antibody. Note the spike and/or dot formations at the periphery of the cells. The closed box is magnified in the inset. Original magnification, ×400, ×600 (inset). Scale bar = 5 μm. Right: the numbers of focal adhesion (FA) detected by vinculin immunoreactivity are presented as means ± SDs. Thirty cells of KO#3, #4, and #31 and mock cells, respectively, were analyzed. Statistical analyses were carried out using the Mann–Whitney *U*‐test. *, *P* < 0.01; **, *P* < 0.005. (B) Migration rate measured using a Transwell assay. Left: the OV‐EBP‐KO and mock cells (m) were seeded in 24‐well Transwell plates and incubated for 24 h in medium without serum. Cells (indicated by arrows) were stained with HE and counted using a light microscope. Scale bar = 10 μm. Right: the numbers of migrated cells are presented as means ± SDs. The experiments were performed in triplicate. Statistical analyses were carried out using the Mann–Whitney *U*‐test. *, *P* < 0.05. (C) Left: wound‐healing assay with OV‐EBP‐KO and mock cells (m). A scratch ‘wound’ was introduced to the middle of wells containing cells grown to confluency, and phase contrast images were taken after 9, 12, and 15 h. Scale bar = 50 μm. Right: the values of wound areas in 0 h were set as 1. The fold wound areas are presented as means ± SDs. The experiments were performed in triplicate. Statistical analyses were carried out using the Mann–Whitney *U*‐test. *, *P* < 0.05. (D) Western blot analysis for the indicated proteins in total lysates from OV‐EBP‐KO and mock cells. The experiments were performed in duplicate.

Cell mobility is closely associated with dynamic assembly and disassembly of FA [28, 29]. To further examine the association between FA status and cell mobility, OV‐EBP‐KO and mock cells were stained with anti‐vinculin antibody (a marker of mature FA) [27, 28]. The number of FAs per unit area was significantly higher in OV‐EBP‐KO as compared to mock cells (Fig. 3A). The OV‐EBP‐KO cells also had significantly increased migration rates as compared to the mock cells (Fig. 3B) and refilled wounded empty spaces more rapidly (Fig. 3C). This was accompanied by increased expression of several EMT‐related molecules (including N‐cadherin, vimentin, and Snail), some cancer stem cell (CSC)‐related markers (including ALDH1 and Sox2), and decreased expression and phosphorylation of ERM protein (Fig. 3D), which is a membrane‐cytoskeletal linking protein [30].

**Fig. 4:**
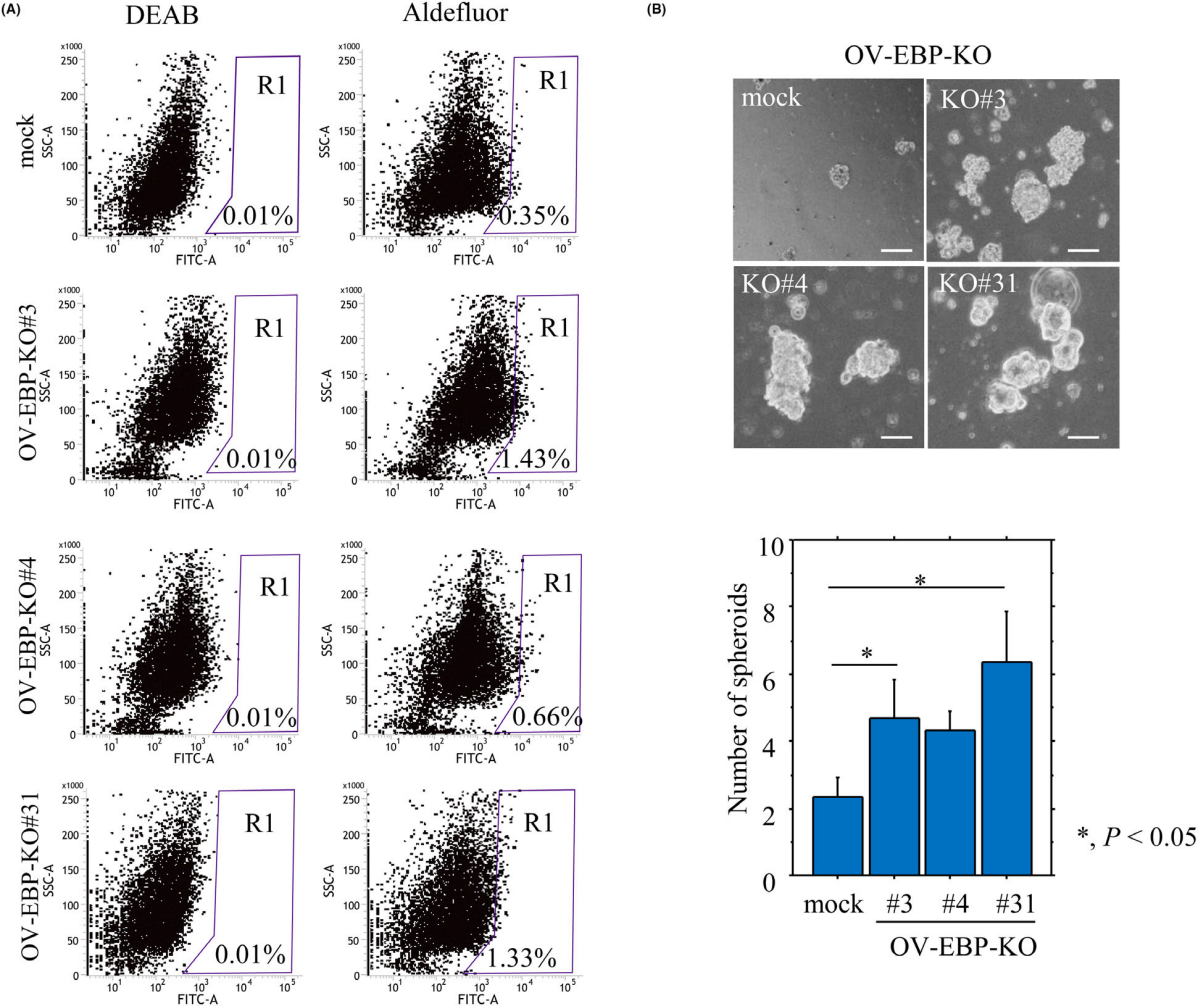
Relationship between membranous EBP50 (Me‐EBP50) knockout (KO) and cancer stem cell (CSC)‐like features. (A) Aldefluor analysis in OV‐EBP‐KO and mock cells. Note the R1 populations including the ALDH^high^ population with CSC‐like features. The experiments were performed in duplicate. (B) Upper: phase contrast photographs of spheroids formed by OV‐EBP‐KO and mock cells following 2 weeks of growth. Scale bar = 50 μm. Lower: the numbers of spheroids are presented as means ± SDs. The experiments were performed in triplicate. Statistical analyses were carried out using the Mann–Whitney *U*‐test. *, *P* < 0.05.

A significant increase in the ALDH^high^ population, including a high percentage of CSC‐like cells, was also observed in the OV‐EBP‐KO cells compared with the mock cells (Fig. 4A). This was consistent with the significant increase in the number of well‐defined, round spheroids > 50 μm in diameter (Fig. 4B).

These findings suggest that Me‐EBP50 KO decreases cell proliferation, accelerates cell motility, and engenders CSC‐like properties through induction of an EMT‐like phenotype in OCCC cells.

### 3.3 Me‐EBP50 strongly interacts with MYH9 in OCCC cells

**Fig. 5:**
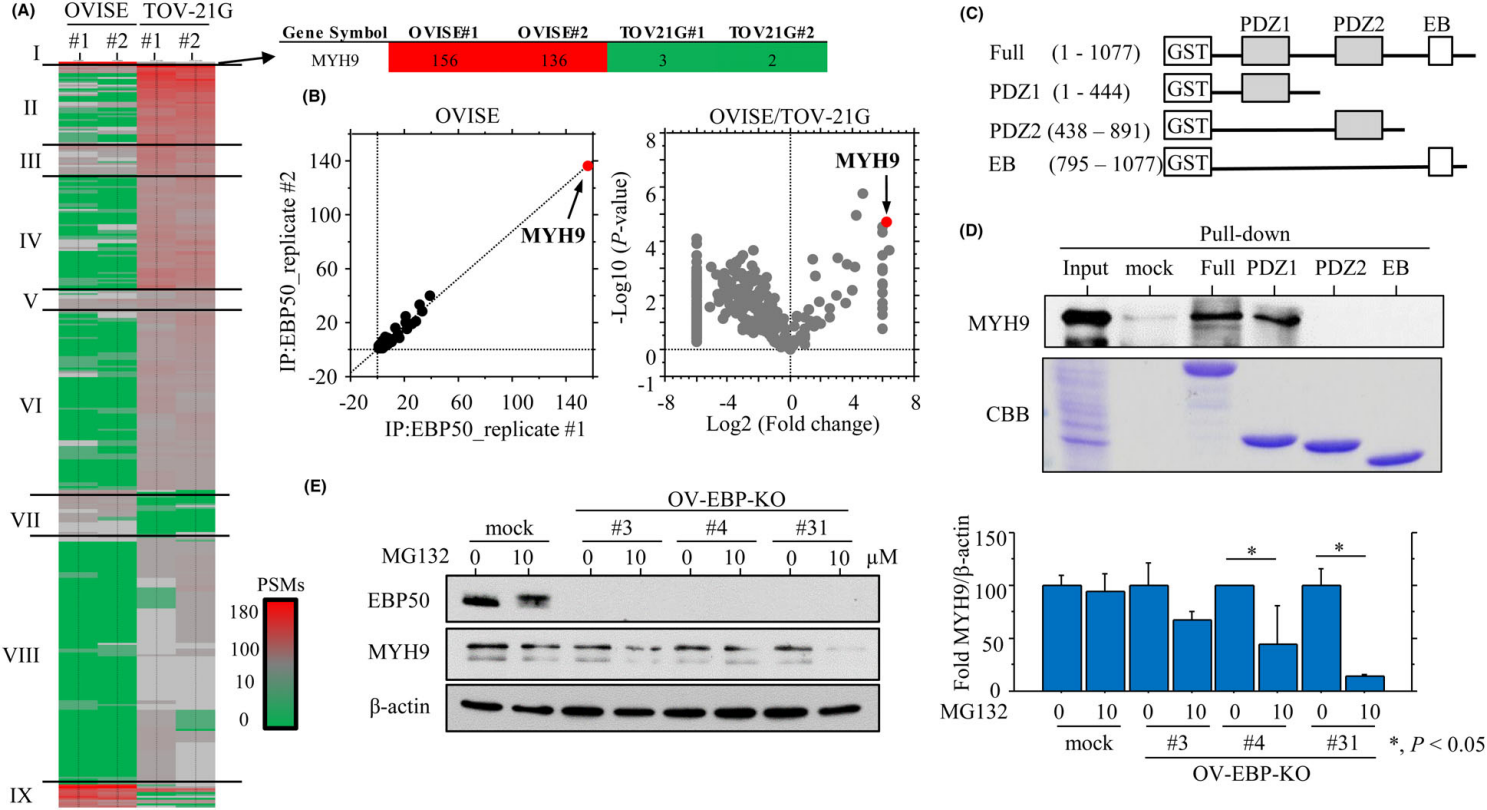
Interaction between EBP50 and myosin 9 (MYH9) in ovarian clear cell carcinoma (OCCC) cells. (A) Unsupervised hierarchical clustering of proteoforms detected by a combination of EBP50‐mediated co‐immunoprecipitation and shotgun proteomics in OVISE and TOV‐21G cells. The values of peptide spectrum matches (PSMs) are color coded as follows; red, gray, and green indicated high (> 100), neutral (10–100), and low (< 10), respectively. Major clusters are shown as groups I to IX. (B) Left: Reproducibility between technical replicates. A scatterplot of proteoforms using TOV‐21G cells and the respective squared Pearson correlation *R* ^2^ are shown. Note that MYH9 has a high PSM score (indicated by arrow). Right: Volcano plots generated to compare the relative PSM values obtained from OVISE relative to TOV‐21G cells. (C) Schematic representation of the cytosolic PSD‐95/*Drosophila* discs large/ZO‐1 (PDZ) and EB domains of EBP50. (D) Upper: Proteins bound to the beads were analyzed followed by western blot analysis for MYH9 in OVISE cells. Lower: Detection of GST‐bound EBP protein by Coomassie Brilliant Blue (CBB). The experiments were performed in duplicate. (E) Left: western blot analysis for the indicated proteins in total lysates from OV‐EBP‐KO and mock cells treated with 10 μm MG132 for 24 h. Right: The values of endogenous MYH9 protein expression detected by western blot were normalized to β‐Actin. The fold changes in protein expression are presented as means ± SDs. The values of signals in vehicle‐treated cells (0 μm) were set as 100. The intensity of individual signals was measured using imagej software version 1.41 (NIH, Bethesda, MD, USA; http://imageJ.nih.gov/ij). The experiments were performed in triplicate. Statistical analyses were carried out using the Mann–Whitney *U*‐test. *, *P* < 0.05.

EBP50 is characterized by at least two PDZ molecules that bind the C‐terminus of target proteins in a sequence‐specific manner [31]. To identify proteins associated with Me‐EBP50, we carried out shotgun proteomics on proteins that co‐immunoprecipitated with EBP50, and found a total of 271 and 791 proteoforms in OVISE and TOV‐21G cells, respectively. As shown in Fig. 5A, hierarchical clustering separated these proteins into nine groups. Of these, group I included MYH9 proteoforms with high peptide spectrum matches (PSMs) from OVISE cells; this was in contrast to the low values in TOV‐21G cells. There was high reproducibility between the technical replicates in OVISE cells (Pearson correlation *R* ^2^ of 0.9). A volcano plot, which represents each proteoform as a relative PSM calculated as the Log2 (fold‐change) and the −Log10 (*P*‐value), also revealed that MYH9 was an outlier (Fig. 5B). These observations indicate that MYH9 was strongly enriched in Me‐EBP50 co‐immunoprecipitates in OVISE cells. To further map the MYH9‐binding region within EBP50, we performed GST pull‐down assays with full length and truncated forms of EBP50 (Fig. 5C). This revealed that MYH9 specifically bound to GST‐EBP50‐PDZ1 (but not PDZ2 and EB domains; Fig. 5D). In addition, treatment with MG132 (nonspecific proteasome inhibitor) revealed the half‐life of MYH9 protein to be significantly decreased in OV‐EBP‐KO cells as compared to the mock cells (Fig. 5E). These findings suggest that Me‐EBP50 stabilizes MYH9 post‐translationally, likely via a strong interaction with MYH9 through the PDZ1 domain.

### 3.4 Inhibition of MYH9 leads to induction of EMT features, decreased proliferation, and accelerated mobility in OCCC cells

**Fig. 6:**
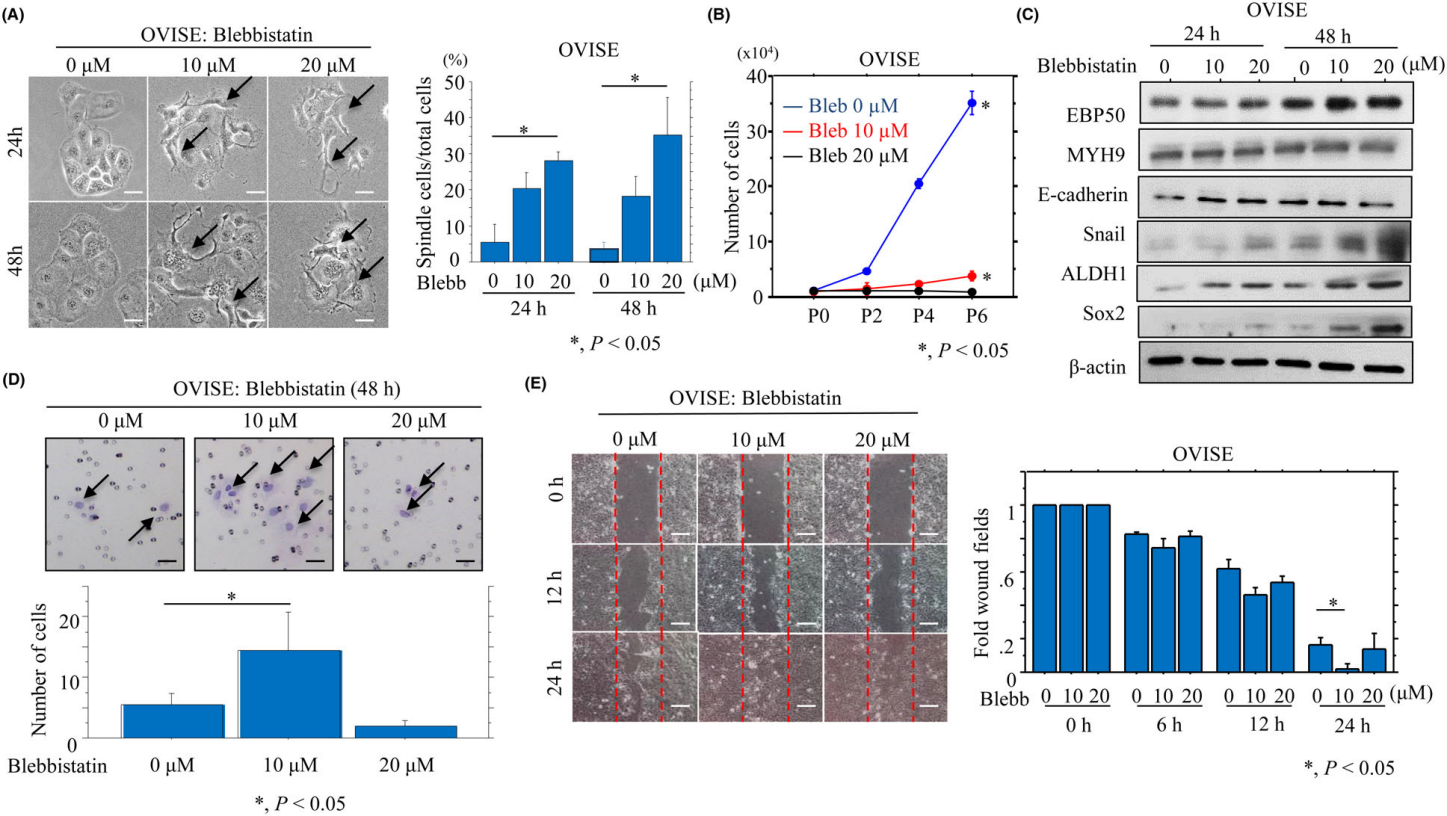
Changes in cell morphology, proliferation, and migration following inhibition of myosin 9 (MYH9) by blebbistatin in ovarian clear cell carcinoma (OCCC) cells. (A) Left: phase contrast images of OVISE cells for the time shown following treatment with 10 and 20 μm blebbistatin (Blebb). Note the switch toward fibroblastic appearances in OVISE cells (indicated by arrows). Scale bar = 10 μm. Right: the percentages of spindle‐shaped relative to total cells are presented as means ± SDs. The experiments were performed in triplicate. Statistical analyses were carried out using the Mann–Whitney *U*‐test. *, *P* < 0.05. (B) OVISE cells were seeded at low density with or without 10 and 20 μm blebbistatin (Blebb). The cell numbers are presented as means ± SDs. P0, P2, P4, and P6 are 0, 2, 4, and 6 days after seeding, respectively. The experiments were performed in triplicate. Statistical analyses were carried out using the Mann–Whitney *U*‐test. *, *P* < 0.05. (C) Western blot analysis of the indicated proteins in OVISE cell lysates after 10 and 20 μm blebbistatin treatment for the times shown. The experiments were performed in duplicate. (D) Migration rate measured using a Transwell assay. Upper: OVISE cells were seeded in a 24‐well Transwell plates and incubated in medium without serum after 10 and 20 μm blebbistatin treatment for 24 h. Cells (indicated by arrows) were stained with HE and counted using a light microscope. Scale bar = 10 μm. Lower: the number of migrated cells is presented as means ± SDs. The experiments were performed in triplicate. Statistical analyses were carried out using the Mann–Whitney *U*‐test. *, *P* < 0.05. (E) Left: wound‐healing assay with OVISE cells following 10 and 20 μm blebbistatin (Blebb) treatment. A scratch ‘wound’ was introduced to the middle of wells containing cells grown to confluency, and phase contrast images were taken after 12 and 24 h. Scale bar = 50 μm. Right: the values of wound areas in 0 h were set as 1. The fold wound areas are presented as means ± SDs. The experiments were performed in triplicate. Statistical analyses were carried out using the Mann–Whitney *U*‐test. *, *P* < 0.05.

Since EBP50 exerts its biological functions through interaction with other molecules [31], we evaluated the role of its binding partner, MYH9, using blebbistatin, a synthetic chemical compound that effectively and reversibly blocks MYH9 ATPase activity [32]. Treatment of OVISE cells with blebbistatin dramatically altered morphology toward a fibroblastic appearance in a dose‐dependent manner (Fig. 6A), along with a significant decrease in proliferation (Fig. 6B), increased expression of Snail, ALDH1, and Sox2 (Fig. 6C), and a significant increase (at 10 μm but not 20 μm blebbistatin) in migration (Fig. 6D,E). The effects were also enhanced in blebbistatin‐treated OV‐EBP‐KO cells as compared to the mock cells (Fig. S2). These findings suggest that suppression of MYH9 also affects EMT features, proliferation, and mobility in OCCC cells, and are consistent with the results we obtained with Me‐EBP50‐KO OVISE cells.

### 3.5 High co‐expression of Me‐EBP50 and MYH9 is associated with a favorable prognosis in OCCC

**Fig. 7:**
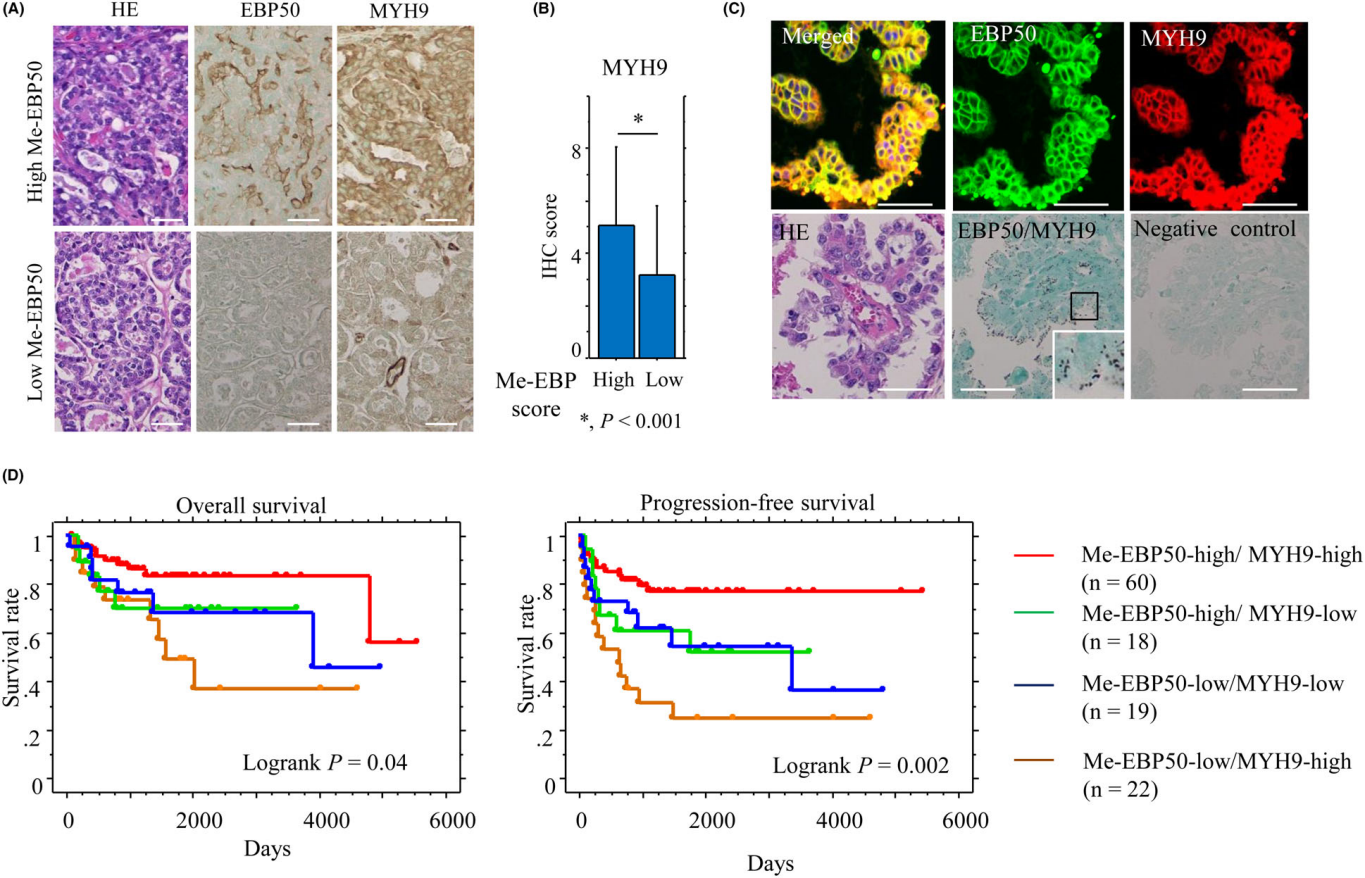
Relationship between expression of membranous EBP50 (Me‐EBP50) and myosin 9 (MYH9) and prognosis in ovarian clear cell carcinoma (OCCC). (A) Staining by HE (left) and immunohistochemistry (IHC) for EBP50 (middle) and MYH9 (right) in OCCC. Original magnification, ×200. Scale bar = 50 μm. (B) IHC scores for MYH9 between high and low Me‐EBP50 categories. The data shown are means ± SDs. A total of 120 OCCC cases were immunohistochemically investigated. Statistical analyses were carried out using the Mann–Whitney *U*‐test. *, *P* < 0.001. (C) Upper: immunofluorescence for EBP50 and MYH9 in OCCC. Note the co‐immunolocalization of EBP50 and MYH9 at the plasma membrane. Original magnification, ×100. Ten OCCC cases were examined. Lower: PLA assay for the EBP50/MYH9 interaction in OCCC. Note the small aggregated dots in apical plasma membrane compartments of the tumor cells. The closed box is magnified in the inset. Original magnification, ×200 and ×400 (inset). Scale bar = 50 μm. Five OCCC cases were investigated. (D) OS (left) and PFS (right) relative to combined Me‐EBP50 and MYH9 (a combination of high and low scores). *n*, number of cases.

Representative IHC images for Me‐EBP50 and MYH9, as well as E‐cadherin, Snail, ALDH1, Vimentin, and Sox2 are illustrated in Fig. 7A and Fig. S3A. MYH9 score was significantly higher in the Me‐EBP50‐high category as compared to the Me‐EBP50‐low group (Fig. 7B), whereas Snail and ALDH1 scores were significantly lower in the former (Fig. S3B), consistent with the finding that Me‐EBP50 score was positively correlated with MYH9 score and inversely correlated with Snail score (Table S1). Co‐immunolocalization of EBP50 and MYH9 was observed at membranous components of OCCC cells, in line with the results of a PLA assay, which can be used for quantification of protein–protein interactions in cells or tissue sections [33], demonstrating interactions between the two molecules at apical plasma membranous sites (Fig. 7C).

High Me‐EBP50 expression was significantly associated with early clinical stage and a lack of distant metastasis (Table 1). Kaplan–Meier analysis showed that a combination of high Me‐EBP50 and high MYH9 scores was associated with the longest OS and PFS as compared to the other combinations (Fig. 7D), although there was a lack of association between MYH9 status and prognosis (Fig. S4). Univariate Cox progression hazards regression revealed that Me‐EBP50, as well as several clinicopathological factors, were significant prognostic indicators for OS and PFS. Multivariate Cox regression analysis also showed that Me‐EBP50, FIGO stage, and tumor peritoneal dissemination were independent prognostic factors for PFS but not OS (Table 2).

**Table 1:** Relationship between Me‐EBP50 immunoreactivity and clinicopathologic factors in ovarian clear cell carcinomas. FIGO, International Federation of Gynecology and Obstetrics; LN, lymph node; Me, membranous; *n*, number of cases; pT, factor refers to the criteria of the TNM classification; pT, pathological tumor stage.

	*n*	Me‐EBP50 immunoreactivity [*n* (%)]		*P*‐value
		Low (< 4)	High (≥ 4)	
Age (years)				
< 60	71	21 (29.6)	50 (70.4)	0.3
≥ 60	48	19 (39.6)	29 (60.4)	
FIGO stage				
I	70	19 (12.9)	51 (87.1)	0.004
II	14	7 (50.0)	7 (50.0)	
III	29	9 (31.0)	20 (69.0)	
IV	5	5 (100.0)	0 (0.0)	
pT factor				
1	75	21 (28.0)	54 (72.0)	0.1
2	16	8 (50.0)	8 (50.0)	
3	27	11 (40.7)	16 (59.3)	
LN metastasis				
Positive	14	7 (50.0)	7 (50.0)	0.2
Negative	86	25 (29.1)	61 (70.9)	
Distant metastasis				
Positive	5	5 (100.0)	0 (0.0)	0.003
Negative	115	34 (30.4)	78 (69.6)	
Dissemination				
Positive	21	10 (47.6)	11 (52.4)	0.2
Negative	98	30 (30.6)	68 (69.9)	

**Table 2:** Univariate and multivariate analysis for overall survival and progression‐free survival in ovarian clear cell carcinoma. Empty cells indicate not applicable. FIGO, International Federation of Gynecology and Obstetrics; LN, lymph node; Me, membranous; pT, pathological stage. pT factor refers to the criteria of the TNM classification.

Variable	Univariate analysis				Multivariate analysis		
	Cutoff	Log‐rank value	*P*‐value	Favorable factor	Hazard ratio	95% CI	*P*‐value
Overall survival							
Me‐EBP50	3/4	4.7	0.02	High level	0.6	0.23–1.8	0.4
MYH9	5/6	0.7	0.3				
Age	59/60	0.6	0.4				
FIGO stage	I, II/III, IV	47.4	< 0.001	III, IV	0.1	0.03–0.81	0.02
pT factor	1/2,3	30	< 0.001	2, 3	0.9	0.26–4.21	0.9
LN metastasis	Positive/negative	4.7	0.02	Negative	2.02	0.44–9.22	0.3
Distant metastasis	Positive/negative	22.2	< 0.001	Negative	0.3	0.04–2.2	0.2
Dissemination	Positive/negative	43.9	< 0.001	Negative	0.1	0.03–0.88	0.03
Progression‐free survival							
Me‐EBP50	3/4	10.4	0.001	High level	0.3	0.14–0.66	0.02
MYH9	5/6	1.4	0.2				
Age	59/60	0.5	0.4				
FIGO stage	I,II/III,IV	74.3	< 0.001	III, IV	0.2	0.08–0.88	0.03
pT factor	1/2,3	32.9	< 0.001	2, 3	0.8	0.31–2.27	0.7
LN metastasis	Positive/negative	18.6	< 0.001	Negative	0.5	0.17–1.83	0.3
Distant metastasis	Positive/negative	34.5	< 0.001	Negative	0.2	0.04–1.23	0.08
Dissemination	Positive/negative	57.8	< 0.001	Negative	0.2	0.06–0.67	0.008

## 4 Discussion

The present study clearly provides the evidence that patients with expression of Me‐EBP50, which localizes at the apical plasma membrane of polar epithelia, were significantly associated with favorable prognosis for both OS and PFS in OCCC as compared to that of Cyt‐EBP50 expression. High Me‐EBP50 expression was also significantly associated with early clinical stages and a lack of distant metastasis, consistent with the idea that apically localized EBP50 is required to maintain epithelial polarity, which is a morphological characteristic disrupted early in the development of epithelial malignancies [34, 35]. Moreover, Me‐EBP50‐KO decreased expression and phosphorylation of ERM protein; this is in line with a report that EBP50 plays an important role in the maintenance of active ERM protein at the cortical brush border membrane of polarized epithelia [36]. By contrast, Cyt‐EBP50 translocates to the nucleus and interacts with PARP1 in response to several survival factors. This leads to increased PARP1 activity and inhibition of apoptosis, which in turn increases the likelihood of tumor recurrence [23]. The opposite effects of EBP50 in OCCC are in line with evidence that EBP50 is tumor suppressive when localized underneath the plasma membrane, but oncogenic when localized in the cytoplasm or nucleus, or when deleted [17, 31, 37, 38].

We also found that Me‐EBP50‐KO induced a fibroblast‐like morphology in OCCC cells, increased expression of some EMT‐related markers, reduced cell proliferation, accelerated migration capability, and induced CSC‐like properties. Me‐EBP50‐KO also increased the number of FAs distributed at the cell periphery, in line with acceleration of cell migration capability, whereas EBP50‐null vascular smooth muscle cells had fewer and larger FAs than wild‐type cells [28]. The discrepant results may be due to differences in cell types or subcellular localization of EBP50. In addition, the expression of EMT/CSC‐related markers including Snail and ALDH1 was significantly higher in the Me‐EBP50‐low category relative to the Me‐EBP50‐high group in OCCC tissues; these findings corroborate reports of migratory cells having a lower proliferation rate than cells in the tumor core, and that EMT promotes stem cell properties and further generates cells with CSC‐like features [39, 40, 41, 42]. Given that the particular cell architecture of EBP50‐depleted cells is suggestive of a switch to a migratory phenotype characterized by the appearance of structures such as lamellipodia, which are involved in cell migration [43, 44, 45], we suggest that Me‐EBP50 inhibits migration capability through modulation of the EMT program in OCCC cells. In addition, it has been reported that EBP50 overexpression inhibits Matrix Metalloproteinase‐2 (MMP‐2) activity and that knockdown of EBP50 promotes MMP‐2 activity in breast and uterine cervical carcinoma cells, suggesting that EBP50 inhibits metastasis via suppression of MMP‐2 activity [46].

Several lines of evidence from our study indicate that Me‐EBP50 functionally interacts with MYH9 in OCCC cells. First, a combination of co‐immunoprecipitation and shotgun proteomics revealed that Me‐EBP50 binds strongly to MYH9 in OCCC cells; this is consistent with the co‐immunolocalization and interaction of EBP50 and MYH9 at the apical plasma membrane in OCCC cells, and a positive correlation between IHC scores of the two molecules in OCCC tissues. Second, the inhibition of MYH9 by blebbistatin phenocopied the results we obtained in the Me‐EBP50‐KO cells, leading to an induction of EMT‐like phenotype and acceleration of migration. The effects were further enhanced in blebbistatin‐treated Me‐EBP50‐KO cells, suggesting that Me‐EBP50 may facilitate MYH9 organization, which is required for regulation of migration capability [26]. Third, the half‐life of MYH9 protein was significantly decreased in Me‐EBP50‐KO cells as compared to mock cells, prompting us to speculate that Me‐EBP50 may post‐translationally stabilize MYH9. Finally, we demonstrated that EBP50 directly interacted with MYH9 through its PDZ1 domain. Given that MYH9 is required for maintenance of the equilibrium between acto‐myosin and microtubule networks that controls cell motility [47, 48, 49], we suggest that modulation of the cytoskeleton may require Me‐EBP50 and its association with MYH9. In support of this conclusion, disorganization of the microtubule‐MYH9 network after EBP50 depletion triggers microtubule polymerization, Rac1 activation, formation of lamellipodia, and the migration of vascular smooth muscle cells [43]. A loss of Me‐EBP50 expression may also be due to alterations in several EBP50‐related signal pathways associated with cell mobility [17, 19, 31, 50, 51].

Unexpectedly, we found that OV‐EBP‐KO cells expressed E‐cadherin and several EMT‐related molecules. Given our data showing a frequent co‐expression of E‐cadherin and vimentin in OCCC tissues, it is possible that they are in a hybrid, epithelial–mesenchymal (E/M) stage [52]. A similar hybrid stage was also described in a long‐term primary culture of human OCCC cells [53]. Interestingly, cancer stemness seems to be associated with a partial EMT phenotype rather than full‐blown EMT [54, 55, 56], in line with our results that OV‐EBP‐KO cells could induce CSC‐like properties.

Finally, Me‐EBP50 expression was an independent favorable prognostic factor for PFS but not OS in OCCC, although no such association was observed in the case of MHY9. However, the best prognosis for OS and PFS was found in OCCC patients with a combination of Me‐EBP50‐high and MYH9‐high scores, suggesting that a combined IHC analysis of EBP50 and MYH9 may have great utility in OCCC prediction and prognosis. In fact, EBP50 directly or indirectly affects tumor behavior through interaction with binding partners and the subsequent modulation of downstream signaling pathways [57, 58].

## 5 Conclusion

**Fig. 8:**
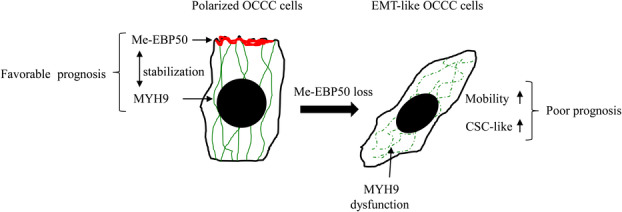
Schematic representation of the interplay between membranous EBP50 (Me‐EBP50) and myosin 9 (MYH9) and prognosis in ovarian clear cell carcinoma (OCCC). Functional interaction between Me‐EBP50 and MYH9 at the apical plasma membrane polarizes OCCC cells (left), whereas loss of Me‐EBP50 induces an epithelial–mesenchymal transition (EMT)‐like phenotype through MYH9 dysfunction, leading to increased mobility and cancer stem cell (CSC)‐like properties (right).

Our results suggest a novel role of Me‐EBP50 in OCCC (Fig. 8). Apical Me‐EBP50 exerts a tumor suppressor function by establishing and maintaining epithelial polarization through modulation of the MYH9‐dependent cytoskeleton network. In contrast, loss of Me‐EBP50 expression and the consequent MYH9 dysfunction could induce an EMT‐like phenotype, resulting in increased cellular mobility and enhanced CSC‐like features, which in turn promote OCCC progression. Together, our data show that EBP50 expression and localization are clinically relevant biomarkers in OCCC patients.

## Conflict of interest

The authors declare no conflict of interest.

## Author contributions

MN, TM, and MS carried out the majority of the experiments, analyzed the data, and wrote the manuscript. They were helped by AY, MH, YO, RK, YI, TI, KO, YH, NF, and YK. All authors reviewed and approved the final manuscript.

### Peer review

The peer review history for this article is available at https://www.webofscience.com/api/gateway/wos/peer‐review/10.1002/1878‐0261.13503.

## Supporting information


**Fig. S1.** Differences in expression of several molecules and migration capacity between OVISE and TOV‐21G cells.

**Fig. S2.** Changes in cell morphology, proliferation, and migration following inhibition of MYH9 by blebbistatin in OV‐EBP‐KO cells.

**Fig. S3.** Relationship between expression of EBP50 and EMT/CSC‐related markers in OCCC.

**Fig. S4.** Relationship between MYH9 expression and prognosis in OCCC.

**Table S1.** Correlation between expression of Me‐EBP50 and the related molecules in ovarian clear cell carcinomas.

## Data Availability

The data that support the findings of this study are available on request from the corresponding author.
