# Supplementary figures and images for: Interaction between membranous EBP50 and myosin 9 as a favorable prognostic factor in ovarian clear cell carcinoma
Source: Mol Oncol. 2023 Aug 30;17(10):2168–82. doi: 10.1002/1878-0261.13503 (PMC10552901; doi:10.1002/1878-0261.13503)

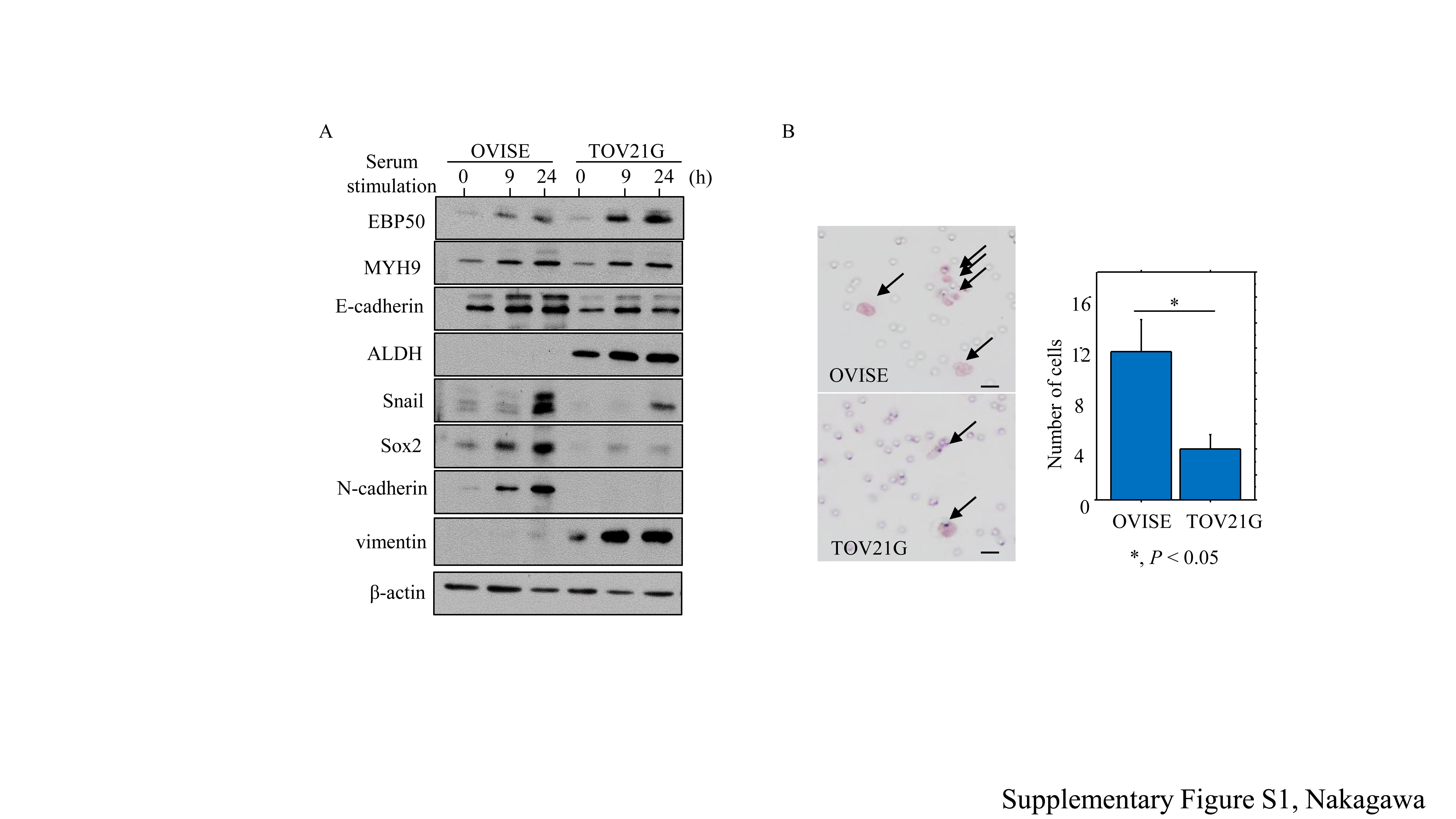

Supplement: Supplementary file 1 — Fig. S1. Differences in expression of several molecules and migration capacity between OVISE and TOV‐21G cells. [file MOL2-17-2168-s002.tif]

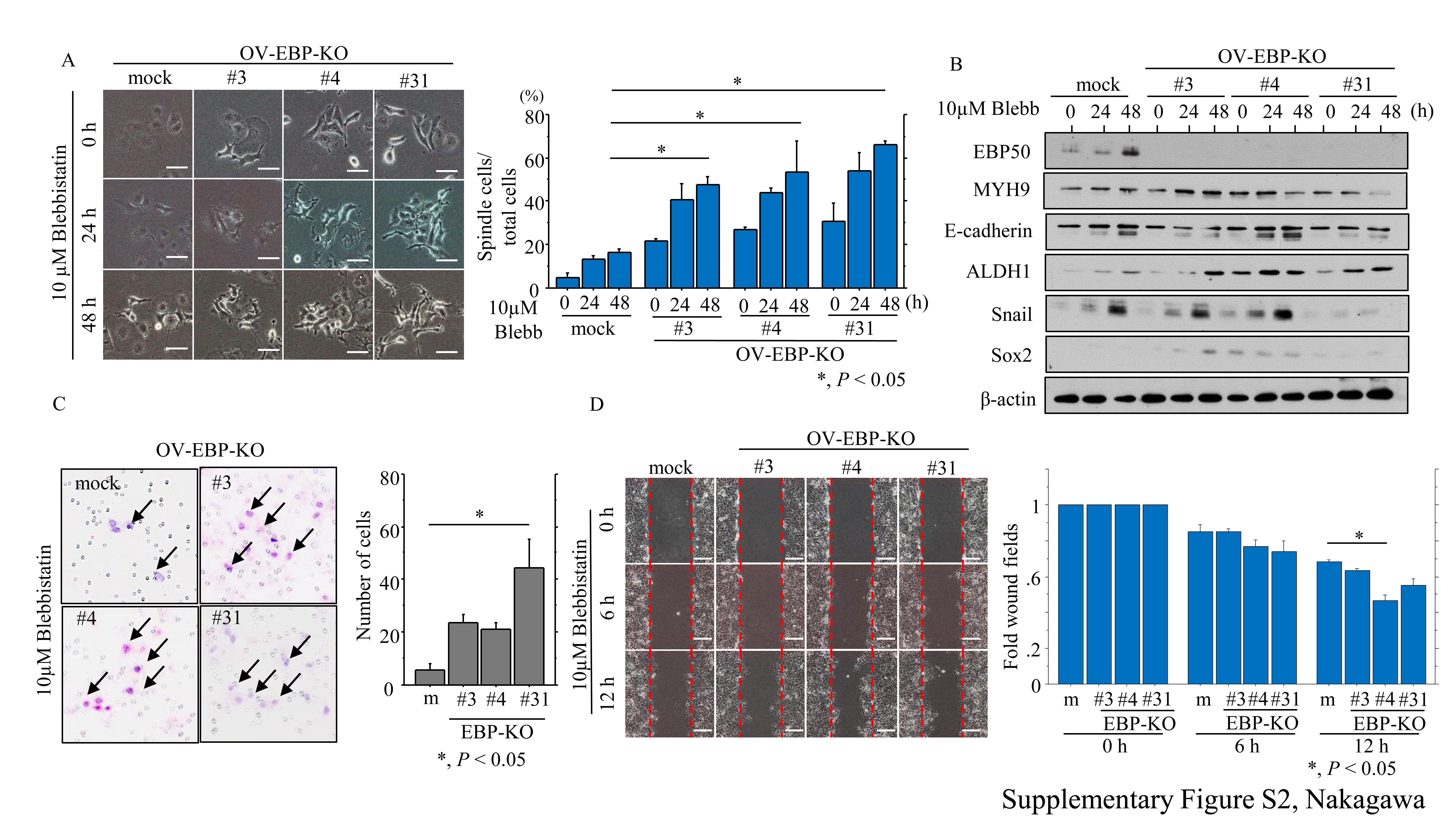

Supplement: Supplementary file 2 — Fig. S2. Changes in cell morphology, proliferation, and migration following inhibition of MYH9 by blebbistatin in OV‐EBP‐KO cells. [file MOL2-17-2168-s004.tif]

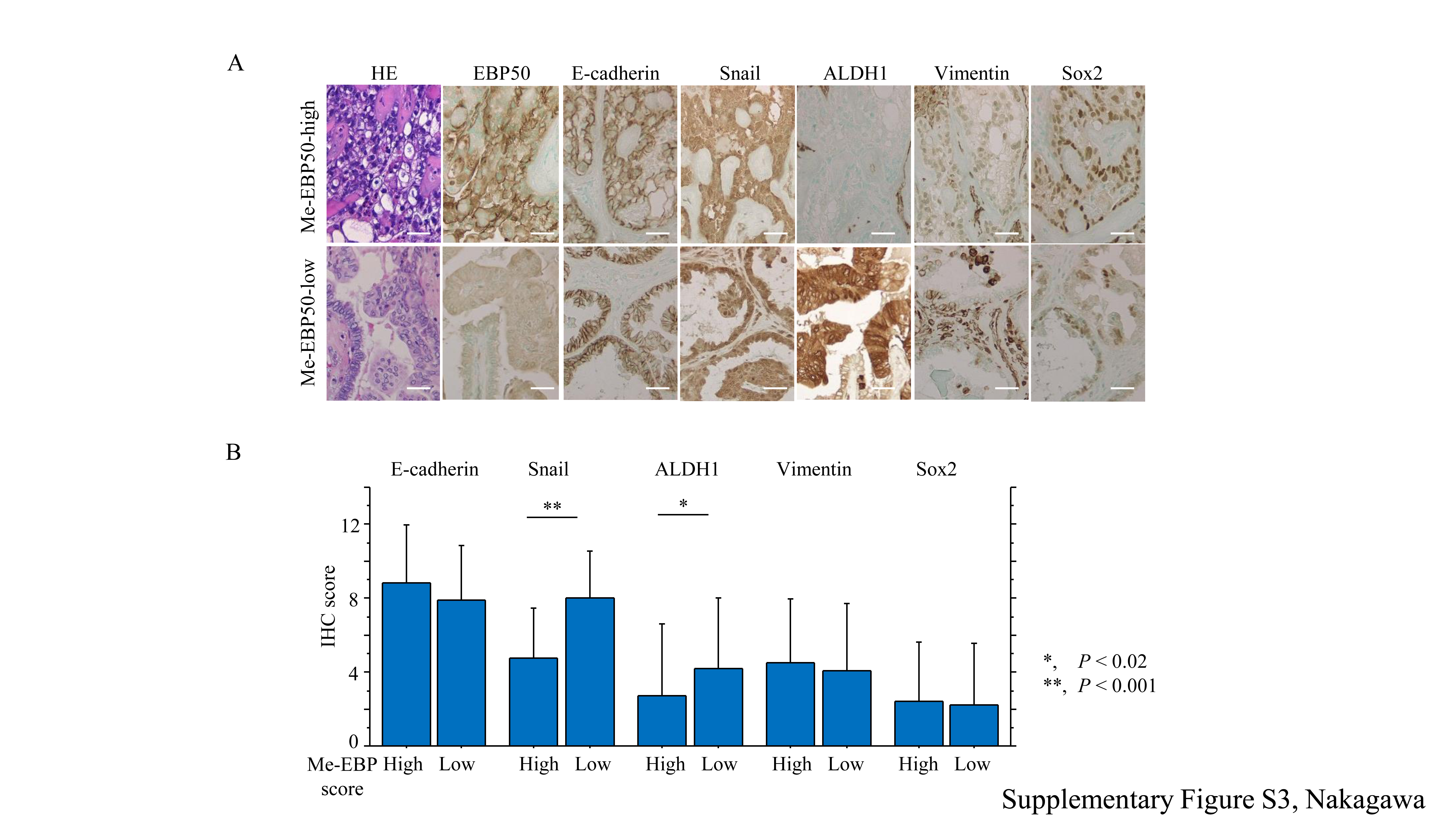

Supplement: Supplementary file 3 — Fig. S3. Relationship between expression of EBP50 and EMT/CSC‐related markers in OCCC. [file MOL2-17-2168-s005.tif]

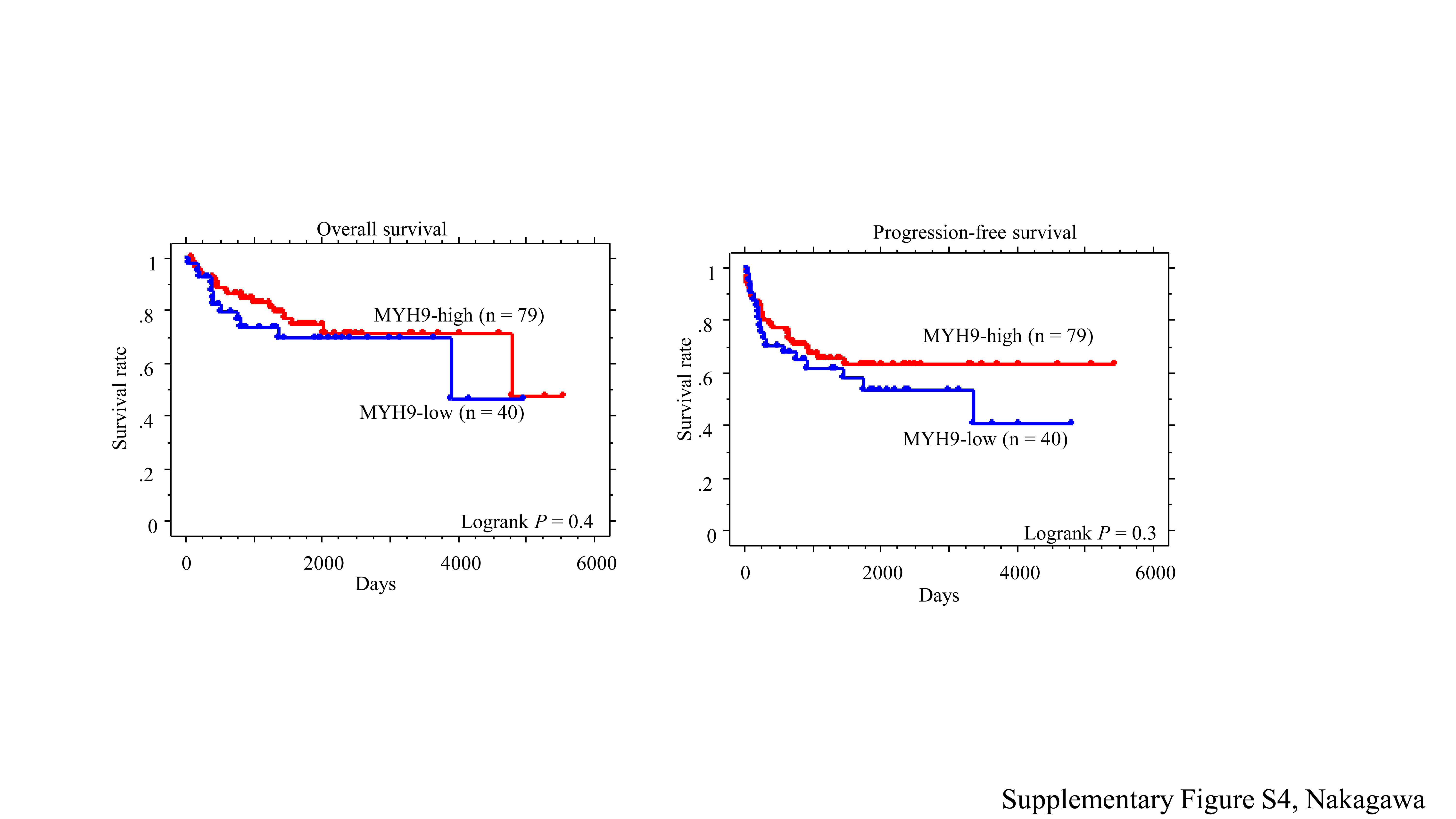

Supplement: Supplementary file 4 — Fig. S4. Relationship between MYH9 expression and prognosis in OCCC. [file MOL2-17-2168-s003.tif]
